# Multimethod Assessment of Mentalizing and its relations with Somatic Symptoms in Adolescents with Primary Headache

**DOI:** 10.1192/j.eurpsy.2022.1122

**Published:** 2022-09-01

**Authors:** F. Bizzi, S. Charpentier Mora, M. Tironi, A. Riva, R. Nacinovich

**Affiliations:** 1 University of Genoa, Department Of Educational Sciences (disfor), Genoa, Italy; 2 San Gerardo Hospital, Child Neuropsychiatry Clinic, Monza, Italy

**Keywords:** mentalizing, Primary Headache, Adolescents, somatic symptoms

## Abstract

**Introduction:**

Difficulties in mentalizing (i.e., the ability to reflect on self and others’ internal mental states, operationalized as reflective functioning [RF]; Fonagy et al., 2012) have been associated with psychological symptoms (Luyten et al., 2020), including somatic symptoms (Bizzi et al., 2019). Therefore, the assessment of its dimensions may be clinically relevant for young patients with somatic symptoms, as with Primary Headache (PH), representing one of the most common somatic complaints in children and adolescents.

**Objectives:**

This study aimed to assess RF with a multi-method approach, exploring its relation with somatic symptoms.

**Methods:**

48 adolescents diagnosed with PH (M_age_=14.83, SD=2.81; 67% females) were recruited from an Italian Child Neuropsychiatry Clinic. RF was measured both through the Child and Adolescent Reflective Functioning (CRFS) applied to the Child Attachment Interview transcripts and the self-report Reflective Functioning Questionnaire (RFQ), while the Children’s Somatization Inventory (CSI-24) was used to measure the perceived severity of somatic symptoms.

**Results:**

Different relations with somatic symptoms depended on the method used to evaluate RF: no significant correlations were found with the CRFS subscales (General, Other, Self ), while a negative significant correlation was found with the RFQ subscale Certainty about mental states (RFQ_C) (r=-.46, p=.016). All subscales of CRFS were negatively correlated with RFQ_C (p=.05), but not with the other RFQ subscale (Uncertainty about mental states; RFQ_U).

**Conclusions:**

This suggests that two measures may lead to different dimensions of the same construct, thus a multi-method assessment of RF would be advisable in clinical practice.

**Disclosure:**

No significant relationships.

